# Prediction of spontaneous ureteral stone passage: Automated 3D-measurements perform equal to radiologists, and linear measurements equal to volumetric

**DOI:** 10.1007/s00330-017-5242-9

**Published:** 2018-01-24

**Authors:** Johan Jendeberg, Håkan Geijer, Muhammed Alshamari, Mats Lidén

**Affiliations:** Department of Radiology, Faculty of Medicine and Health, Örebro University, Örebro, Sweden

**Keywords:** Computed tomography, Ureteral calculi, Kidney stone, Ureter, Renal colic

## Abstract

**Objectives:**

To compare the ability of different size estimates to predict spontaneous passage of ureteral stones using a 3D-segmentation and to investigate the impact of manual measurement variability on the prediction of stone passage.

**Methods:**

We retrospectively included 391 consecutive patients with ureteral stones on non-contrast-enhanced CT (NECT). Three-dimensional segmentation size estimates were compared to the mean of three radiologists’ measurements. Receiver-operating characteristic (ROC) analysis was performed for the prediction of spontaneous passage for each estimate. The difference in predicted passage probability between the manual estimates in upper and lower stones was compared.

**Results:**

The area under the ROC curve (AUC) for the measurements ranged from 0.88 to 0.90. Between the automated 3D algorithm and the manual measurements the 95% limits of agreement were 0.2 ± 1.4 mm for the width. The manual bone window measurements resulted in a > 20 percentage point (ppt) difference between the readers in the predicted passage probability in 44% of the upper and 6% of the lower ureteral stones.

**Conclusions:**

All automated 3D algorithm size estimates independently predicted the spontaneous stone passage with similar high accuracy as the mean of three readers’ manual linear measurements. Manual size estimation of upper stones showed large inter-reader variations for spontaneous passage prediction.

**Key points:**

• *An automated 3D technique predicts spontaneous stone passage with high accuracy.*

• *Linear, areal and volumetric measurements performed similarly in predicting stone passage.*

• *Reader variability has a large impact on the predicted prognosis for stone passage.*

## Introduction

Ureteral stones are one of the most common causes of acute flank pain, with large and increasing costs for the health care [1, 2]. Earlier studies [3–7] have shown that about 80% of ureteral stones pass spontaneously into the urinary bladder. In the absence of complications, if the pain is manageable and the stone can be expected to pass within a reasonable time without surgical intervention, the first approach is conservative, with radiological and clinical surveillance [8]. If a stone is not expected to pass it is usually treated with extracorporeal shock wave lithotripsy (ESWL), laser lithotripsy or, in some cases, with percutaneous stone extraction.

To select an appropriate treatment strategy for each individual, prediction of the probability for spontaneous stone passage is important [9].

The correlation between stone size and position and the probability for spontaneous stone passage is strong [3–5, 7], but at present there is no international consensus on a standardized method of stone size measurement with non-contrast-enhanced computed tomography (NECT).

The levels of uncertainty in ureteral stone size measurement are threefold.

First, there are several different opinions on which dimension should be used for measuring a ureteral stone. The width [3, 4], largest axial diameter [5], axial area [10], volume, using either a formula of an ellipsoid or 3D software reconstruction [10, 11], and length as well as the largest size on coronal [12–14], axial and sagittal images have all been proposed. There are also controversies concerning how this dimension should be defined [15].

Second, there are diverging opinions about in which window setting this measurement should be performed and a lack of standardised post processing parameters [13, 15, 16].

The third level of uncertainty is the large intra- and inter-individual differences of stone measurements among radiologists [7, 17, 18], where the implementation and user friendliness of the electronic callipers may influence the reader variations.

In a recent study a regression model using the stone size and location for predicting spontaneous stone passage was introduced [7]. The regression model eliminates the first level of uncertainty through a clear definition of stone measurement and subsequently the second level by using consistent post-processing parameters, including window settings. However, the third level of uncertainty—the reader variability—remains a challenge when the regression model is applied to the size of a stone, as estimated by a radiologist.

Whereas several studies have shown similar reader variability [17, 18], expressed in millimetres, in the size estimation of urinary stones, no study has, to the best of our knowledge, investigated the impact of the variability on the estimated prognosis for the spontaneous passage of a stone.

The use of an automated 3D segmentation of a urinary stone serves two purposes: First, several different dimensions of a stone can be measured, such as the length, width, cross-sectional area and circumference, volume and surface area. [15] Second, by automating the size estimation, the reader variability is eliminated.

The first objective of the present study was therefore to apply a 3D segmentation on a large cohort of ureteral stones to compare the ability of different size estimates to predict spontaneous passage of ureteral stones

The second objective was to investigate the impact of manual measurement variability on the predicted probability of spontaneous stone passage, using the previously published predictive regression model [7].

## Materials and Methods

This retrospective study was approved by the Regional Research Ethics Board who waived informed consent.

### Inclusion and exclusion

**Fig. 1:**
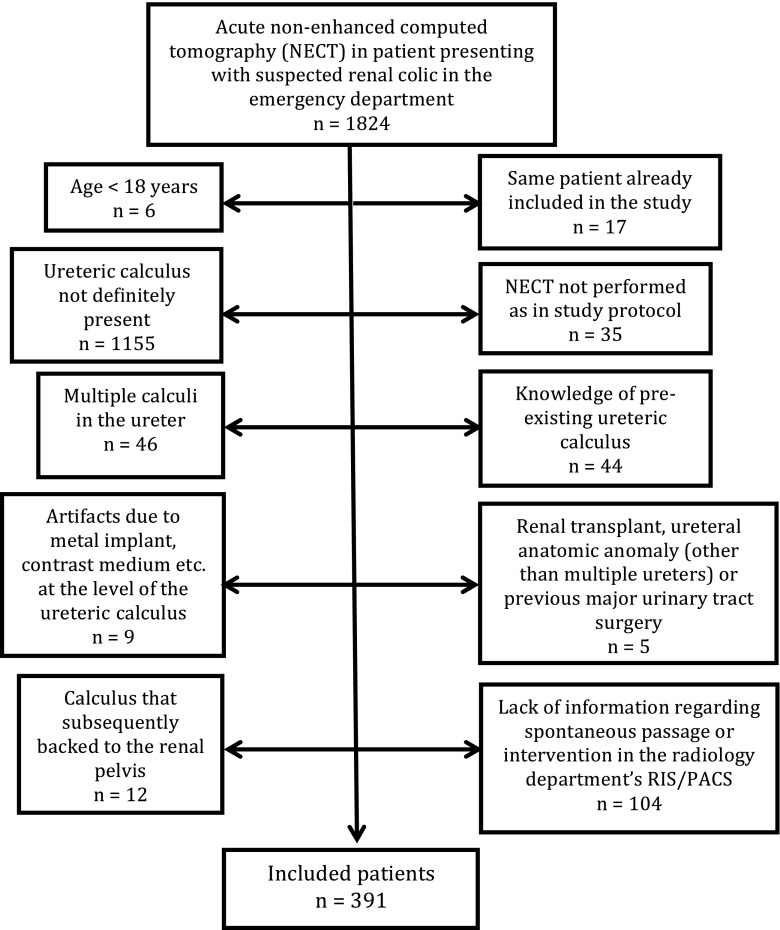
Flowchart of exclusion criteria and numbers

A retrospective review of patients presented at our emergency department who underwent NECT because of acute flank pain in the period from April 2012 to September 2014 yielded 1824 subjects with completed NECT. The inclusion criterion was a solitary ureteral stone > 2 mm in diameter in the axial plane. Exclusion criteria are shown in Fig. 1. From the initial 1824 patients 391 fulfilled the inclusion and exclusion criteria. The same patient cohort was used as in the previous study where predictive stone passage regression curves were created and where further details of the inclusion can be found [7]. One examination among the 392 included patients in the previous study did not include a stack of 1-mm slices and was excluded in the present study.

### CT protocol

The CT examinations were performed on two different CT scanners, either a 40-detector-row CT scanner (Brilliance, Philips Medical Systems) with a low-dose NECT protocol for the urinary tract (120 kV, 70 mAs/slice, CTDI 4.9 mGy, 40 × 0.625 mm, standard filter [B], supine position) or a 2 × 128-channel scanner (Somatom Definition Flash, Siemens) (120 kVp, 70 mAs/slice, CTDI 4.7 mGy, 128 × 0.6 mm, filter B20f, B25f or I30f, supine position). Three- or five-millimetre axial, coronal and sagittal multiplanar reformats (MPR) in the main axes of the patient were generated and used for manual measurements.

One stack of 1-mm axial slices per examination was generated and exported to an image data bank. This stack was used for the 3D segmentation and not for manual measurement.

### Image review

#### Manual measurements

Three radiologists independently measured each ureteral stone with the integrated PACS measurement callipers (Sectra IDS7).

The largest in-plane diameter of the stones was measured on the axial, coronal and sagittal reformats in a bone window (L300/W1120) and in a soft tissue window (L50/W400) [7, 19]. The length was defined as the largest of these measurements and the width as the smallest [7, 15].

#### Automated 3D-segmented measurements

**Fig. 2:**
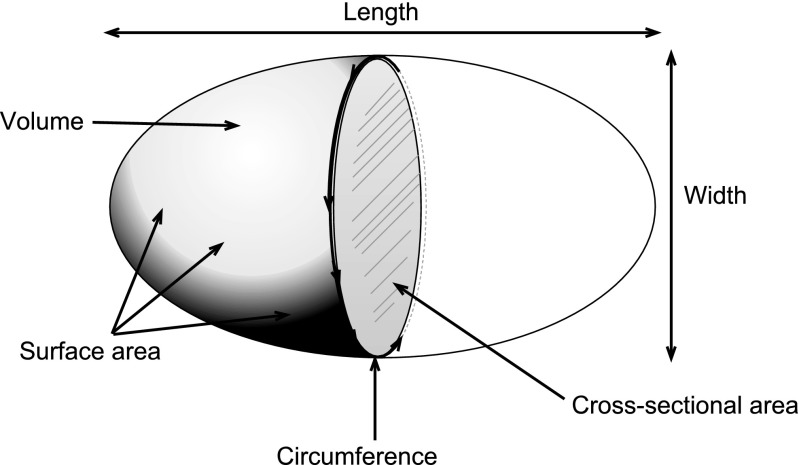
Schematic drawing of the automated measurements of the ureteral stones

An automated segmentation algorithm was developed in Matlab R2016a (Mathworks Inc.) for the study to obtain reader-independent 3D size estimates for the ureteral stones. The segmentation algorithm consisted of three steps: First, the stone and the surrounding tissue in the 1-mm data sets were resampled at 0.25-mm isotropic voxel size. Subsequently, the stones were segmented using simple thresholding, with the threshold defined as one half of the maximum attenuation value in the stone, with a lower limit of 200 Hounsfield units. The lower limit was introduced to avoid inclusion of image noise in the segmented volume. Third, a morphological dilatation with a spherical structuring element with a 2-pixel radius (0.5 mm) was applied to the segmented stone. The structuring element was used to minimise the bias compared to the manual size estimate using the bone window. The length of the stone was defined as the largest distance between two border pixels. The width, circumference and cross-sectional area were computed using automated MPR perpendicular to the long axis of the stone; see Fig. 2. [15]. The surface area and volume of the stone were computed using an alpha shape encompassing all the segmented voxels [ref. http://mathworks.com/help/matlab/ref/alphashape.html].

### Outcome measure—spontaneous passage of stone

We reviewed all radiological examinations in the local RIS/PACS regarding ureteral stone passage or intervention up to 6 months after the initial diagnostic examination. Observed stone passage was defined as the presence of a follow-up radiological examination [CT or intravenous urography (IVU)] where a ureteral stone was definitely not present anymore. If there was stone passage under conservative treatment it was defined as spontaneous passage.

Follow-up subgroups of 4 weeks (± 2 weeks) and 20 weeks were defined for the outcome spontaneous passage of the ureteral stone. The outcome measures were identical with the previous study using the same patient cohort [7].

### Analysis of the impact of inter-reader variability on the predicted outcome of a ureteral stone

#### Main analysis

Using the predictive regression model for the stone width measured in the bone window for upper and lower ureteral stones, which was developed in an earlier study [7], the predicted probability for stone passage was calculated for each of the three readers’ manual measurements separately. The highest and lowest estimated probabilities for each stone were recorded and the difference in percentage points between these probabilities was calculated.

#### Secondary analysis

The inter-reader variability had a large impact on the predicted outcome in upper, but not in lower stones. Therefore, a secondary analysis, similar to the main analysis, was performed for upper stones with the corresponding predictive regression models of the stone length in the bone window and the stone length and width in the soft tissue window [7].

**Fig. 3:**
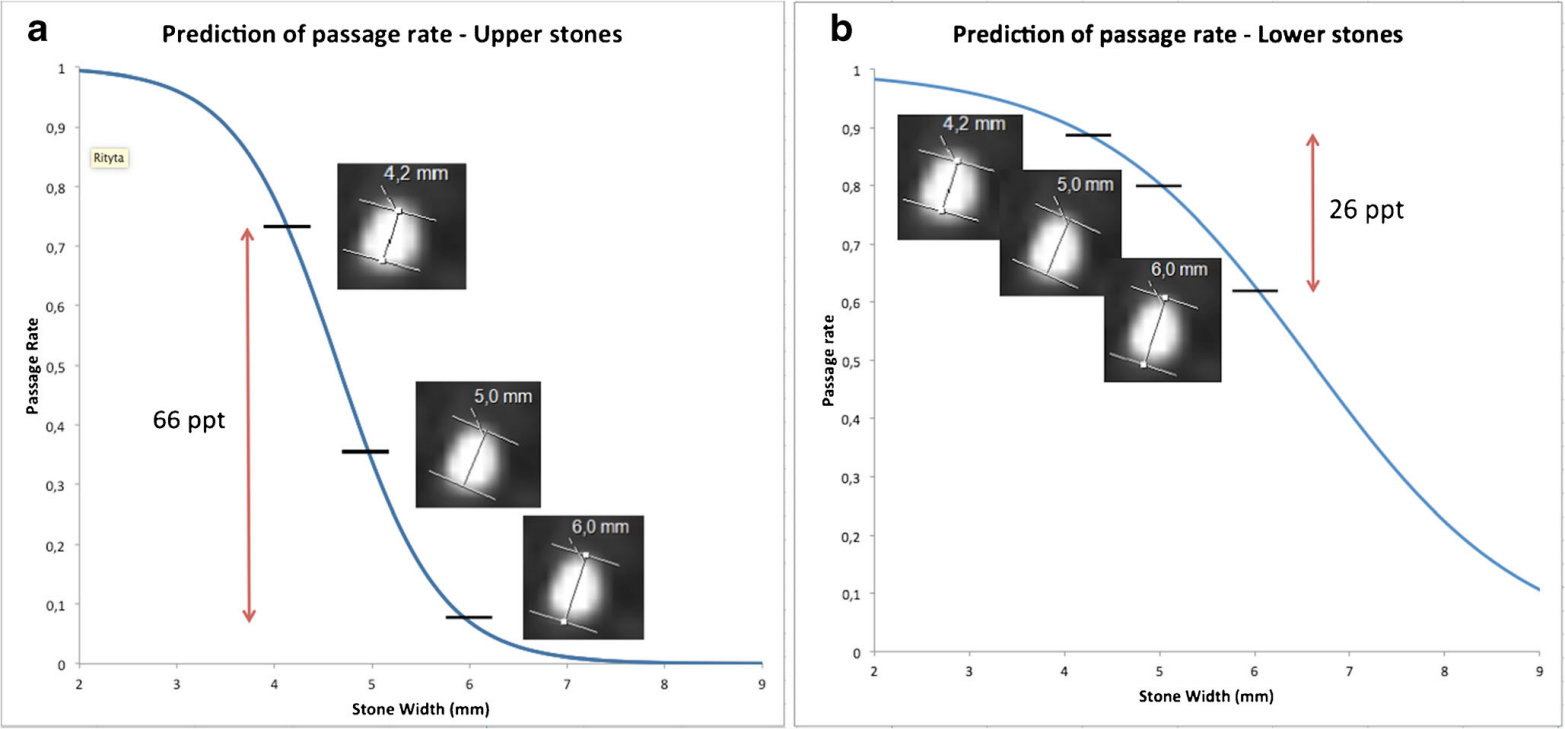
Example of the impact of reader variations on the estimated probability for spontaneous passage within 20 weeks of a ureteral stone. **a**) Upper stones bone window. **b**) Lower stones bone window. NECT of a ureteral stone with three different size estimations in the bone window setting L300/W1120. Upper stone = Cranial to the sacroiliac joint. Lower stone = Overlying or distal to the sacroiliac joint. Double arrow = Difference in the predicted probability of spontaneous passage of the stone between the largest and the smallest manual measurement. ppt = Percentage points. With an estimated size of 4.2 mm, the probability for spontaneous passage within 20 weeks is approximately 73 %, for a 5-mm stone the probability is 35 % and for 6-mm the probability is 7 %. The difference in the predicted probability of spontaneous passage of the stone between the largest and the smallest manual measurement is 66 percentage points

Figure 3 demonstrates an example of one stone with three different size estimates measured in the bone window.

If, for example, the manual stone width estimates in an upper stone for the three readers were 4.2 mm, 5.0 mm and 6.0 mm, the smallest measure would be 4.2 mm and the largest 6.0 mm. These measures were put in the predictive regression model, resulting in a predicted probability for stone passage of approximately 73 % for the 4.2 mm estimate (i.e. 73/100 stones will pass) and of 7 % for the 6.0 mm estimate. This makes a difference in the predicted probability of 66 percentage points. If, on the other hand, the estimates were of a *lower* stone, the predicted probability of spontaneous passage would be 88 % for the 4.2-mm stone and 62 % for the 6-mm stone, giving a difference of only 26 percentage points.

### Statistical analyses

The statistical analysis was performed using IBM SPSS for Mac OS v24.0.0.0 (SPSS Inc.).

ROC curves for the prediction of spontaneous stone passage were generated for the manual measurements length and width and for the automated 3D measurements length, width, circumference, cross-sectional area, surface area and volume. The area under the ROC curve (AUC) with 95% confidence interval was computed for each parameter as an overall measure of the predictive accuracy. The analysis was performed on the whole cohort as well as on the subgroups upper and lower stones according to the position in the ureter. Stones overlying the sacroiliac joint and below were classified as lower stones.

Bland-Altman plots (95% limits of agreement) for the automated 3D vs. average manual measurements from three readers were created for the measures length and width.

The difference in inter-reader variation of predicted probability of stone passage between the four different size estimates in the secondary stone analysis (stone width and length in the bone and soft tissue window, respectively) in upper stones was compared with Friedman’s test.

## Results

The study included 289 (74 %) males and 102 (26 %) females, mean age 50.1 (SD ±16) years (range 18-100). Mean overall stone width was 3.7 (SD ±1.6) mm and 32 % of the stones were located in the upper ureter [mean stone width 4.7 (SD ±1.7) mm] and 68 % in the lower [mean stone width 3.3 (SD ±1.4) mm] in the bone window. Spontaneous stone passage was seen in 311 patients (80 %), 73 (19 %) of the patients underwent an intervention, and 7 patients (2 %) had neither an intervention nor spontaneous passage during the 26-week study period.

### Automated 3D measurements vs. manual measurements

**Fig. 4:**
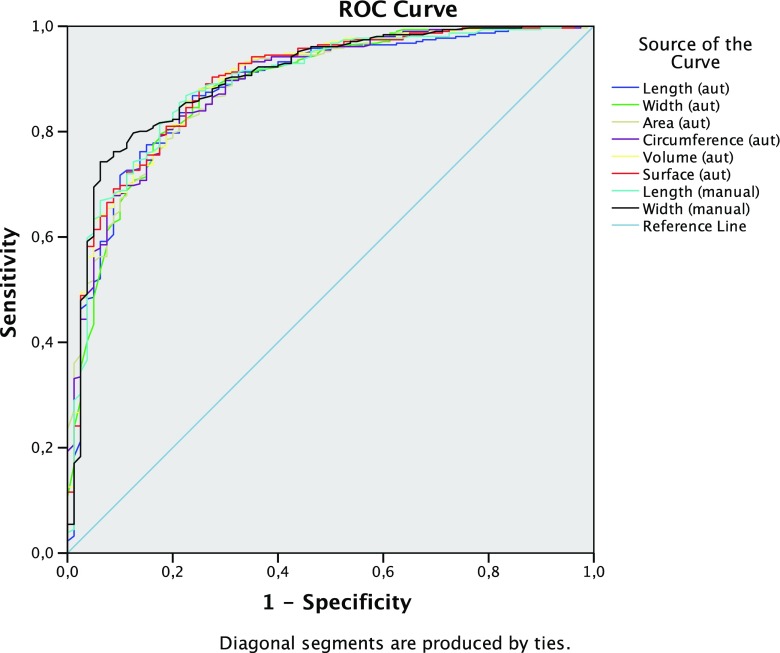
Receiver-operating characteristic (ROC) curves for the prediction of the outcome of spontaneous passage in 20 weeks with eight different measurements. Length = Longest stone axis, width = largest diameter perpendicular to the long axis, area = cross-sectional area perpendicular to the long axis, circumference = circumference perpendicular to the long axis, volume = stone volume, surface = total surface area. (aut) = Automated 3D segmentation-based measurement. (manual) = Mean of three readers’ manual estimations of stone size (bone window)

There were only minimal differences in the area under the curve (AUC) for the various linear, areal and volumetric automated 3D measurements of a ureteral stone in predicting spontaneous stone passage compared to the mean of three readers’ linear manual measurements. As can be seen in Tables 1 and 2 and in Fig. 4, the AUC for all the size measurements in predicting the outcome after 20 weeks ranged from 0.88 to 0.90 in the full cohort, from 0.89 to 0.93 in upper stones and from 0.80 to 0.83 in lower stones.

**Table 1:** Area under the curve (AUC) for the prediction of spontaneous passage of a ureteral stone with different measurements - All stones

Measures	4 weeks			20 weeks		
	AUC	95% CI		AUC	95% CI	
		Lower	Upper		Lower	Upper
Length (aut)	0.85	0.79	0.90	0.88	0.84	0.93
Width (aut)	0.85	0.80	0.91	0.88	0.84	0.93
Area (aut)	0.82	0.75	0.88	0.89	0.85	0.93
Circumference (aut)	0.84	0.77	0.90	0.89	0.85	0.93
Volume (aut)	0.84	0.78	0.90	0.89	0.86	0.93
Surface (aut)	0.85	0.79	0.91	0.90	0.86	0.93
Length (manual)	0.84	0.78	0.90	0.89	0.85	0.93
Width (manual)	0.85	0.79	0.91	0.90	0.86	0.94

Length = Longest stone axis, width = largest diameter perpendicular to the long axis, area = cross-sectional area perpendicular to the long axis, circumference = circumference perpendicular to the long axis, volume = stone volume, surface = total surface area

(aut) = Automated 3D segmentation based measurement. (manual) = Mean of three readers manual estimations of stone size

**Table 2:** Area under the curve (AUC) for the prediction of spontaneous passage of a ureteral stone with different measurements — Subgrouped according to position in the ureter

Measures	Upper stones						Lower stones					
	4 weeks			20 weeks			4 weeks			20 weeks		
	AUC	95% CI		AUC	95% CI		AUC	95% CI		AUC	95% CI	
		Lower	Upper		Lower	Upper		Lower	Upper		Lower	Upper
Length (aut)	0.89	0.81	0.97	0.89	0.83	0.95	0.80	0.70	0.89	0.81	0.71	0.92
Width (aut)	0.89	0.82	0.97	0.90	0.85	0.95	0.80	0.71	0.88	0.80	0.70	0.89
Area (aut)	0.87	0.78	0.96	0.91	0.87	0.96	0.75	0.65	0.86	0.80	0.72	0.89
Circumference (aut)	0.88	0.80	0.97	0.91	0.86	0.96	0.78	0.68	0.87	0.81	0.72	0.89
Volume (aut)	0.90	0.82	0.97	0.91	0.87	0.96	0.78	0.69	0.88	0.82	0.73	0.91
Surface (aut)	0.90	0.82	0.97	0.91	0.86	0.96	0.79	0.70	0.89	0.82	0.73	0.91
Length (manual)	0.89	0.81	0.97	0.89	0.83	0.95	0.79	0.69	0.88	0.83	0.73	0.92
Width (manual)	0.92	0.85	0.99	0.93	0.89	0.97	0.80	0.71	0.89	0.83	0.73	0.93

Length = Longest stone axis, width = largest diameter perpendicular to the long axis, area = cross-sectional area perpendicular to the long axis, circumference = circumference perpendicular to the long axis, volume = stone volume, surface = total surface area

(aut) = Automated 3D segmentation based measurement. (manual) = Mean of three readers manual estimations of stone size

**Fig. 5:**
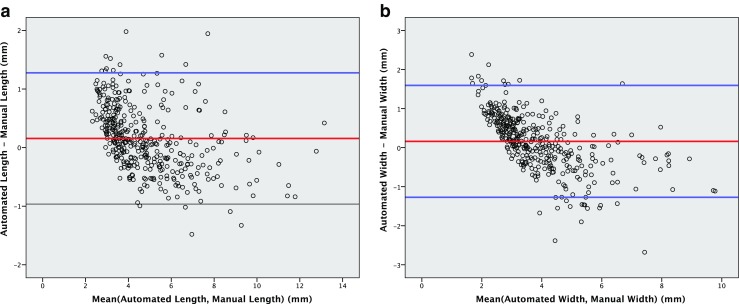
Bland-Altman plots. **a**) Automated length vs. manual length (mean of three readers, bone window). Bland-Altman 95 % limits of agreement 0.2 ± 1.1 mm, *n* = 391. **b**) Automated width vs. manual width (mean of three readers, bone window). Bland-Altman 95 % limits of agreement 0.2 ± 1.4 mm, *n* = 391

The Bland-Altman 95 % limits of agreement between the automated 3D algorithm and the manual measurements (average of three readers) were 0.2 ± 1.1 mm for the stone length and 0.2 ± 1.4 mm for the stone width (Fig. 5). There is a strong tendency towards smaller automatic than manual measurements for larger stones as demonstrated by Fig. 5. This finding is expected since the automatic measurements used a variable threshold defined as one half of the maximum attenuation, whereas the readers used a fixed bone window for measurements. Larger stones have higher peak attenuation resulting in a higher segmentation threshold compared to the smaller stones.

### Impact of inter-reader variability on the predicted outcome of ureteral stones

#### Main analysis

**Fig. 6:**
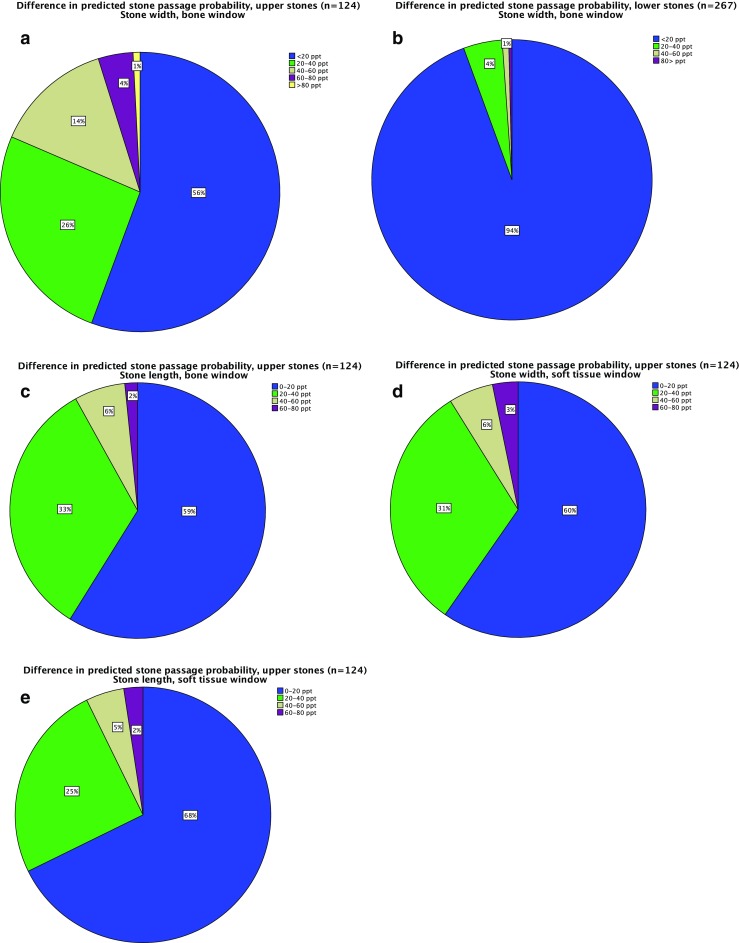
Difference in predicted probability for spontaneous passage based on the largest and smallest manual size estimate. The proportions of the predictions that differ by < 20 percentage points (ppt), 20-40 ppt, 40-60 ppt, 60-80 ppt and > 80 ppt are shown separately. **a**-**b** Main analysis: stone width, measured in the bone window for upper and lower stones. For upper stones and lower stones, there is a difference of > 20 ppt in 44% and in 6% of the predictions, respectively. **c**-**e** Secondary analysis: for upper stones the difference in predicted probability for spontaneous passage based on the largest and smallest manually estimated length and width was calculated in the bone and soft tissue window separately. The smallest impact of the inter-reader variability on the estimated prognosis was seen for the stone length in the soft tissue window

As with all manual size estimation in radiological images, the three readers in the study measured the stones slightly differently. Figures 3 and 6a-b show the impact of the inter-reader variability of manual stone measurement (stone width in the bone window) on the prediction of spontaneous passage of a ureteral stone.

For the same inter-reader variability expressed in millimetres, the difference in predicted probability for spontaneous passage was small in lower stones and large in upper stones. In 94 % of the stones in the *lower* ureter (20-week follow-up), the difference in predicted probability for spontaneous passage was 0-20 percentage points. Only 15 of these 267 stones (6 %) had a larger discrepancy in estimated probability of passage. In contrast, in the upper ureter, 55 out of 124 stones (44 %) had a difference in predicted probability of more than 20 percentage points.

#### Secondary analysis

The impact of the inter-reader variability in upper stones of the manual estimates of the stone length in the bone window and stone width and length in the soft tissue window is displayed in Fig. 6c-e. When measuring the length of upper stones in the soft tissue window, 32 % of the stones had a difference in predicted probability of more than 20 percentage points. The median [inter-quartile range] difference in predicted probability was 17 ppt [4-35 ppt] for the stone width in the bone window, 16 ppt [6-29 ppt] for the stone length in the bone window, 16 ppt [4-27 ppt] for the stone width in the soft tissue window and 12 ppt [5-26 ppt] for the stone length in the soft tissue window. According to Friedman’s test there was a statistically significant difference in the inter-reader variability between the different estimates, *p* = 0.027.

## Discussion

In this study we demonstrated that an automated segmentation algorithm performs similarly to the mean of three readers’ manual measurements in predicting spontaneous ureteral stone passage, that linear size estimates perform similarly to more complicated measurements and that relatively small inter-reader variability in the manual measurements of upper ureteral stones can cause large differences in the predicted probability of stone passage.

Previous studies have shown that spontaneous passage of a ureteral calculus can be predicted with high accuracy with the knowledge of the calculus’ size and location [3, 4, 7]. Since there can be large differences in the probability of stone passage between stones with only 1 or 2 mm differences in size [7], it is of great importance that the measurements are performed consistently between the readers. The first two levels of measurement uncertainty, the dimension of the measurement and the post-processing parameters, can be solved through a consensus on the dimensions and window settings in which a ureteral stone should be measured. For this purpose, we previously presented separate prediction curves for the width and length of a stone with two different window settings of L300/W1120 [19] and L50/W400, where we also used a high grade of magnification. [7]

An analysis of three radiologists’ stone measurements reveals that relatively small inter-individual variations among the three readers’ measurements result in large discrepancies in the predicted probability of spontaneous stone passage. This was particularly apparent in the upper ureter (cranial to the sacroiliac joint), where almost half of the stones had a variation in predicted probability of more than 20 percentage points, when measuring the width in the bone window. The explanation for the discrepancy between the different parts of the ureter is that the predictive curve for upper stones is distinctly steeper than the predictive curve for lower stones, in the stone size interval of a width of 4 to 6 mm, and that a large number of stones in the upper ureter have a size within this interval [7]. The predictive curve in the lower ureter (overlying or caudal to the sacroiliac joint) is flatter, which makes the prediction less vulnerable to reader variations in size estimation. The impact of the inter-reader variability on the estimated prognosis for spontaneous passage could be significantly reduced by measuring the length of the upper ureteral stones in the soft tissue window, most likely because of a smaller part of the stones appearing in an indefinite grey zone. However, even using the stone length in the soft tissue window, almost one third of the stones had a variation between readers of more than 20 ppt.

This observation underlines the importance of the third level of uncertainty: the possible large inter- and intra-individual variability in stone measurement. To reduce this variability several different automated measurements have been proposed [15, 17, 20]. To our knowledge, none of those have been tested for the prediction of spontaneous passage of a ureteral stone.

In this study we showed that an automated 3D segmentation method of measurement for ureteral calculi performed similarly to the mean of three manual measurements, with 95 % limits of agreement of 0.2 ± 1.1 mm for the stone length and 0.2 ± 1.4 mm for the width in the bone window. This can be compared to the inter-reader variability for the same stones with 95 % limits of agreement among the three readers of 0.7 ± 1.3 mm, 0.7 ± 1.3 and 0.1 ± 1.1 mm for the estimation of stone width [7].

The largest differences in AUC for the prediction between the various tested manual and automated dimensions of measurement were seen in the 4-week follow-up of the lower stones subgroup, but even there the AUC only ranged from 0.75 to 0.80. In the total cohort the difference was only 0.03, which we consider to be very small. Consequently, it is of minor importance which of these size estimates we use, but of major importance that we use the chosen estimate consistently. Some authors have recommended the *volume* for the surveillance of stone burden because it is more sensitive to size changes than a linear one-dimensional measurement [17]. For the detection and diagnosis of a ureteral stone the volume is an unnecessarily complicated way of reporting the stone size. In this setting we recommend reporting the length in the soft tissue window as it is intuitive for both the radiologist and the urologist to use, because the predictive strength is similar using linear measures to using the area or the volume of a stone and because the impact of the inter-reader variability in upper stones is smaller compared to the stone width and compared to measurements in the bone window. Nevertheless, the manual measurements are sensitive to variability and we recommend performing measurements using an automated segmentation algorithm.

A relevant future objective would be to integrate an automated segmentation measurement tool in the daily workflow/PACS to help the radiologist perform a consistent review of the stone disease. Together with the stone location, which can also be automatically determined, a semi-automated prediction of the probability for spontaneous ureteral stone passage could be performed with just one click.

There are some limitations to this study. As it was a retrospective study, the follow-up examinations could not be standardised. At the time of the study, our urology department mainly used IVU as a follow-up examination. Obviously there was a risk of missing non-obstructive stones that were either very small or had low density using IVU. However, every radiological examination in the following 26 weeks after that diagnostic NECT was checked for possible missed stones, and we consider the risk of missing clinically significant ureteral stones to be low.

One limitation is that the same cohort of patients that was used for development of the automated measurements also was used for validation against reader size estimations, which can cause a bias towards greater accuracy. Also, the true size of the stones remains unknown. The automated measurements need further validation with another patient cohort and a natural next step would be a prospective study on patients with acute ureteral colic.

A further limitation is that we have not tested different 3D segmentation models against each other and that the 3D segmentation model used in this study is not commercially available. Other approaches, such as semi-automatic algorithms, may further improve the agreement between the mean radiologist measurement and the segmentation algorithm and may therefore be preferable for the prediction of spontaneous stone passage.

In conclusion, our results show that an automated 3D segmentation algorithm of stone measurement (combined with stone location) can predict the spontaneous passage of a ureteral stone with the same high accuracy as the mean of three readers’ manual stone measurements and represents a promising way of eliminating the intra- and inter-individual variability of stone measurements. More complicated measures, such as cross-sectional area or volume, do not increase the predictive accuracy compared to the length or width of a stone. With manual size estimation of upper ureteral stones, the predicted probability for spontaneous passage has large inter-reader variations, whereas the variation in lower ureteral stones is less significant.

## Abbreviations and acronyms

AUC: Area under the curve
ESWL: Extracorporeal shock wave lithotripsy
IVU: Intravenous urography
MPR: Multiplanar reformats
NECT: Non-enhanced computed tomography
PACS: Picture archiving and communication system
ppt: Percentage points
RIS: Radiology information system
ROC: Receiver-operating characteristic
